# Targeting BCL2 pathways in CLL: a story of resistance and ingenuity

**DOI:** 10.20517/cdr.2023.97

**Published:** 2023-11-27

**Authors:** Amanda Reyes, Tanya Siddiqi

**Affiliations:** ^1^Hematology & Oncology, City of Hope, Duarte, CA 91010, USA.; ^2^Hematology/HCT, City of Hope, Duarte, CA 91010, USA.

**Keywords:** BCl-2 inhibitors, apoptosis, CLL, resistance, tumor microenvironments, cell cycle regulation, genetic mutations, epigenetics, richter transformation

## Abstract

Chronic lymphocytic leukemia (CLL) is common amongst leukemic malignancies, prompting dedicated investigation throughout the years. Over the last decade, the treatment for CLL has significantly advanced with agents targeting B-cell lymphoma 2 (BCL2), Bruton’s tyrosine kinase, and CD20. Single agents or combinations of these targets have proven efficacy. Unfortunately, resistance to one or multiple of the new treatment targets develops. Our review investigates various mechanisms of resistance to BCL2 inhibitors, including mutations in BCL2, alterations in the Bcl protein pathway, epigenetic modifications, genetic heterogeneity, Richter transformation, and alterations in oxidative phosphorylation. Additionally, the review will discuss potential avenues to overcome this resistance with novel agents such as bispecific antibodies, Bruton’s tyrosine kinase (BTK) degraders, non-covalent BTK inhibitors, and chimeric antigen receptor T (CART).

## INTRODUCTION

Chronic lymphocytic leukemia (CLL) has been the most common form of Leukemia in the developed world for the last decade, according to the Surveillance epidemiology and end result database^[1]^. Treatment and, therefore, overall prognosis have improved significantly during this time. Investigation into the pathophysiology of CLL allowed for the development of targeted agents, including Burton’s tyrosine kinase (BTK) inhibitors, anti-CD20 monoclonal antibody, and B-cell lymphoma 2 (BCL2) inhibitors^[2]^. Ibrutinib, a BTK inhibitor, proved to be an effective treatment of CLL in the first line^[3]^. Venetoclax, a BCL2 inhibitor, was first utilized in relapsed disease alone and then in combination with rituximab [Table 1]^[4-10]^. More recently, the combination of ibrutinib and venetoclax was approved for front-line treatment of CLL in Europe after findings from the GLOW trial [NCT03462719] and CAPTIVATE trials [NCT02910583]^[6,7]^ [Table 1].

**Table 1:** Venetoclax trials in CLL

**Drug**	**Line of treatment**	**Target**	**Trial**	**Duration of treatment**	**Rate of Richter’s transformation**
Venetoclax + Ibrutinib	First line	BCL2 + BTK	GLOW^[6]^	Fixed	3 patients (2.8%) *vs.* 2 patients (1.9%) in control arm
Venetoclax + Ibrutinib	First line	BCL2 + BTK	CAPTIVATE^[7]^	Fixed	Not documented
Venetoclax + Ibrutinib	Relapsed/Refractory	BCL2 + BTK	CLARITY^[8]^	Fixed	0 patients
Venetoclax	Refractory, 17p mutated	BCL2	Phase II^[9]^	Till progression	11 patients (10.3%)
Venetoclax + Rituximab	Refractory	BCL2 + CD20	MURANO^[5]^	Fixed	6 patients (3.1%) *vs.* 5 patients (2.65%) in control arm
Venetoclax + Obinutuzumab	First line	BCL2 + CD20	CLL14^[10]^	Fixed	2 patients (0.94%) *vs.* 1 patient (0.46%) in control arm

BCL2: B-cell lymphoma 2; BTK: Bruton’s tyrosine kinase; CLL: chronic lymphocytic leukemia.

**Figure 1:**
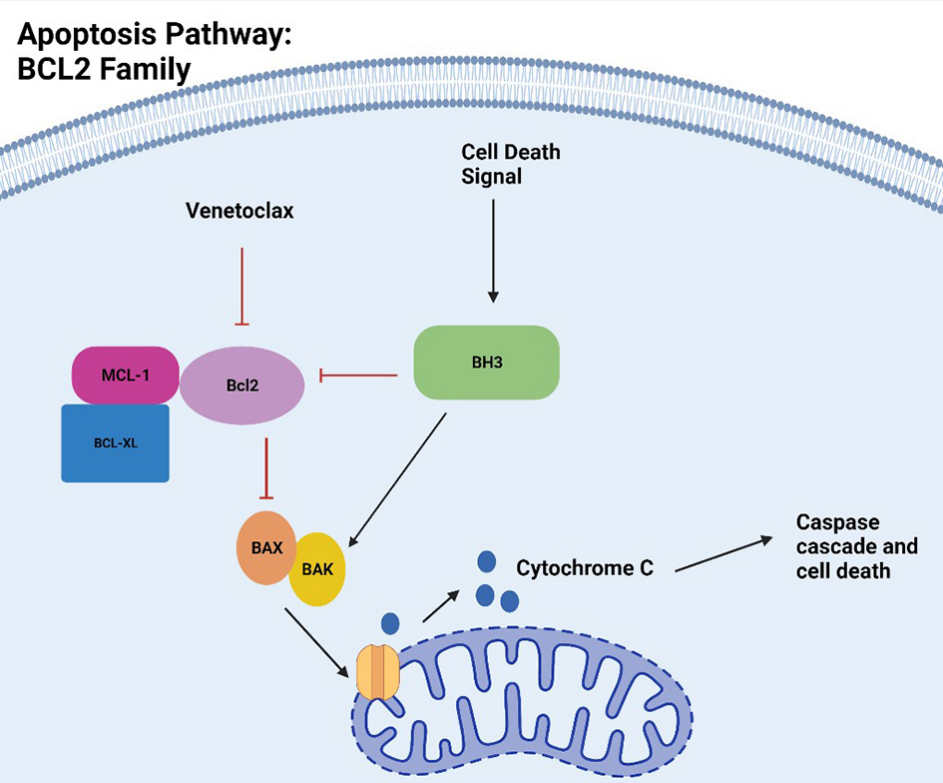
Apoptosis Pathway: BCL2 Proteins. Made with Bioreader with data from the following publications: Roy *et al.*^[11]^; Youle *et al.*^[12]^. BCL2: B-cell lymphoma 2.

Widespread use of venetoclax in hematologic malignancies prompted further research into the BCL22 apoptosis pathway, allowing for the identification of the key agents involved. From extensive research, we have found that in non-cancerous cells, after receiving a pro-apoptotic signal, the BH3-only proteins will activate additional proteins, BAX and BAK, by binding directly or by binding to anti-apoptotic proteins, BCL2, BCL-XL, MCL-1, thereby freeing these pro-apoptotic proteins to travel to the mitochondrial membrane forming pores, releasing cytochrome c which stimulates the caspase cascade for apoptosis^[11,12]^ [Figure 1]. Venetoclax promotes apoptosis by binding to BCL2, enabling the release of the pro-apoptotic proteins to trigger apoptosis^[13]^ [Figure 1]. Unfortunately, resistance to venetoclax develops by several distinct mechanisms, including mutations in BCL2, epigenetic pathways, alterations in oxidative phosphorylation, alterations in BCL2 pathway, tumor microenvironment, genetic heterogeneity, and Ritcher’s transformation. We will discuss each of these mechanisms, focusing on the contributions to resistance in this review. Additionally, we will propose various methods to overcome the various resistance pathways.

## BCL2 INHIBITOR RESISTANCE MECHANISMS

### Genetic mutations in BCL2

Alterations in the substrate or target thereby conferring resistance is a common theme in biology and venetoclax resistance is no exception. A recent analysis of CLL patients who progressed on venetoclax found that 7 of the 15 patients developed a mutation, Gly101Val, in BCL2, which decreases the affinity of BCL2 for venetoclax by overcrowding the BH3 binding groove, thereby preventing venetoclax from displacing the pro-apoptotic proteins [Table 2]^[14-18]^. Of note, the mutation was not detected prior to starting treatment but rather was detected after 19-42 months of treatment^[14]^. Mutations that also confer resistance due to the impact on binding include Phe104lle located at the venetoclax binding site of BCL2 and Asp103Tyr, an essential part of hydrogen binding of venetoclax which have been identified in follicular lymphoma and CLL, respectively^[15,16]^ [Table 2]. In mantle cell lymphoma cell lines, Phe104Cys and Phe104leu missense mutations have also been found to alter the BH3 domain and therefore binding affinity^[17]^ [Table 2]. From a retrospective analysis of CLL patients whose disease was refractory to ibrutinib and resistant to venetoclax, multiple mutations including point mutations in Gly101Ala, Ala113Gly, Leu119Val, Asp113Glu and in-frame insertion of Arg107_Arg110 were observed^[18]^ [Table 2]. Of note, the BCL2 mutations were noted to be sub-clonal with a varying percentage of cells (from 7%-70%, vast majority < 50%), indicating multiple resistance patterns are likely involved^[14-18]^. This finding would argue against these mutations representing so-called “driver mutations”, but there is not enough evidence to definitively determine this. As the general CLL population is not tested for the above mutations given their rarity, it is impossible to give an overall frequency. From the original study identifying the G101V mutation, 21 out of 67 patients had progression on venetoclax, of which 15 samples were analyzed and roughly 50% (7 patients) developed the mutation after venetoclax as the mutation was not present prior^[14]^. With further analysis into venetoclax-resistant patients, additional mutations conferring various changes in the structure of the BCL2 will likely be identified and methods to overcome these mutations will follow.

**Table 2:** Venetoclax resistance: mutations in BCL2

**Mutation**	**Mutation type**	**Mutation site**	**Found in**	**Frequency in patients**
Gly101Val^[14]^	Point	BH3 binding groove	CLL	7/15 (46.6%)
Phe104lle^[16]^	Point	BH3 binding groove	Follicular lymphoma	1/1 (100%)
Gly101Ala^[18]^	Point	BH3 binding groove	CLL	1/11 (9%)
Asp103Tyr^[15]^	Point	BH3 binding groove	CLL	1/4 (25%)
Ala113Glu^[18]^	Point	Non-binding	CLL	1/11 (9%)
Phe104Cys^[17]^	Point	BH3 binding groove	Murine human-like MCL cell lines	NA
Phe104Leu^[17]^	Point	BH3 binding groove	Murine human-like MCL cell lines	NA
Leu119Val^[18]^	Point	Unknown	CLL	1/11 (9%)
Arg107_Arg110^[18]^	Frame shift	Unknown	CLL	3/11 (27.3%)

BCL2: B-cell lymphoma 2; CLL: chronic lymphocytic leukemia.

### Epigenetic modifications

For the last decade, scientists investigated modifications of translation with gene activation or deactivation and the corresponding downstream effects. In the case of the BCL2 pathway, these epigenetic alterations may play a larger role in resistance than direct mutations in the BCL2 protein. A recent study used advanced molecular techniques including CRISPR, whole-exome sequencing, and methylated DNA immunoprecipitation sequencing to identify a regulatory CpG island within the PUMA (a BH3-only protein) promoter site which was shown to be methylated and therefore silenced gene expression (favoring oxidative phosphorylation and cell survival) after the administration of venetoclax, indicating resistance^[19]^. This data was obtained from both CLL patients (6 patients) and VEN/S63845 resistant cell lines. Further proof of this concept was demonstrated by the restoration of venetoclax function (cell death) after inhibition of methyltransferases^[19]^.

Non-coding RNAs, including microRNA (miRNA) and long non-coding RNA (lncRNA), have been investigated extensively in the last two decades in CLL due to their involvement in cell cycle regulation among other cellular mechanics, thereby promoting resistance^[20-22]^. Additionally, RNA cytosine methyltransferases NSUN1 and NSUN2 have been shown to induce venetoclax resistance in leukemic cells via interactions with RNA polymerase II extension complex, knocking down NSUN1 or NSUN2 returned sensitivity to venetoclax^[23]^. Much is still not fully understood regarding these complex epigenetic regulations, as this is an area of future research and investigation.

### Alterations in BCL2 pathway

As the BCL2 pathway is complex, alterations or upregulation of other components have also been the subject of investigation. Mutations in the effector proteins BAX/BAK may be venetoclax specific as one analysis found mutations in BAX followed venetoclax treatment in 30% of the patients but not after treatment with ibrutinib^[24]^. Further, a mutation in the C terminal transmembrane domain (G179E) of BAX prevents the anchoring of BAX to Mitochondria, thereby blocking venetoclax-induced apoptosis^[17,25]^.

In a 2011 study of the novel agent ABT-737, which inhibits BCL2, BCL-XL, and BCL-w, the levels of MCL-1 and BFL-1 were significantly higher than BCL2 in the population resistant to the drug, while the sensitive population had the highest levels of BCL2 comparatively^[26]^. Conversely, in some tumor models, cells express low levels of BCL2 but are still highly sensitive to BCL-2 inhibition, indicating that the BCL2 protein is a small part of a more intricate process^[17]^. Other anti-apoptotic proteins, such as MCL-1 and BCL-XL, are not directly inhibited by venetoclax but appear to have a role in resistance, with BCL-XL appearing to have the strongest impact^[27]^. The overexpression of BCL-XL is associated with venetoclax resistance and the upregulation of NF-kB signaling (cell survival); the addition of BCL-XL inhibitors can restore cell sensitivity to venetoclax^[27]^.

Likewise, MCL-1, involved in the sequestration of BIM and binding of BAK which prevents apoptosis is commonly overexpressed in venetoclax-resistant patients^[13,28]^. MCL-1 has been the subject of interest as there are various efforts at utilizing its inhibition as a potential therapeutic option, occasionally in conjunction with venetoclax^[29]^. Yet still, the matter is more complicated as additional proteins involved in this pathway were also found to have interactions with MCL-1 and BCL-XL, namely BFL-1^[30]^. Research into the detailed interactions between the pro-apoptotic and anti-apoptotic protein signaling balance is warranted, as any number of these proteins could be targeted for treatment.

### Tumor microenvironment

For added complexity, tumor microenvironment including alterations in cell metabolism and signaling may also contribute to resistance. When CLL and MCL cells were preincubated with anti-apoptotic/pro-growth signaling factors from outside the direct BCL2 pathway, namely sCD40L, IL-10, CpG-ODN, B-cell-activating factor (BAFF), CXCL3, the combination of sCD40L, IL-10, and CpG-ODN had the lowest level of ibrutinib/venetoclax induced cytotoxicity indicating resistance^[31]^. Even high levels of ibrutinib and venetoclax did not achieve an adequate level of cytotoxicity, but when NF-kB signaling was inhibited by the addition of proteasome inhibitors, bortezomib and carfilzomib, sensitivity to ibrutinib/venetoclax returned^[31]^. Further research into this topic may reveal potential therapeutic targets for patients experiencing relapse/resistance.

### Genetic heterogeneity

The extensive variation in the genetics of CLL patients has been noted, and the most common alterations include deletions of chromosomes 13q, 11q, 17p, and trisomy 12^[32]^. This heterogeneity may also play a role in resistance, as seen with the 17p deletion, which is not only associated with advanced disease/poor prognosis but also correlated with resistance, as one study found 7 out of 11 venetoclax-resistant CLL patients harbored the *TP53* aberration^[18,32]^. Further, trisomy 12 contributes to increased expression of MCL-1 which also has been associated with venetoclax resistance^[33]^. In addition to chromosomal alterations, other genetic alterations have been associated with resistance; in a study of 8 venetoclax resistance patients, 2 patients were found to have potential targetable mutations (BRAF and PD-L1), both thought to be involved in MCL-1 upregulation^[34]^. Additionally, a homozygous mutation in *CDKN2A/B*, a cell cycle regulator, was also identified in the resistant patient population^[34]^. A combined analysis of several CLL studies found *TP53*, *SF3B1*, *MYD88*, *NOTCH1*, and *ATM* were the most mutated genes with varying rates of mutation across the studies^[32]^. Interestingly, there was variation across the mutations, as certain mutations arise continuously throughout disease (*TP53*, *ATM*), others arise after treatment initiation (*NOTCH1*), while others remain in the same frequency throughout the disease course (*MYD88*)^[35]^.

### Abnormal oxidative phosphorylation

As the BCL2 pathway ultimately involves mitochondria, there has been consideration of the role of oxidative phosphorylation in resistance. In other cancerous cell lines, increased levels of oxidative phosphorylation and reactive oxygen species are associated with resistance to chemotherapeutic agents^[36]^. Investigation into CLL cell lines in vitro found that the resistant cells had significantly higher levels of both basal and maximal oxygen consumption from ATP production by oxidative phosphorylation as well as increased mitochondrial membrane potential^[37]^. Additionally, after the cell lines were treated with venetoclax, a decline in oxygen consumption was observed, but this was dependent on the ability of pore formation (BAX/BAK) as knockout cell lines did not have the same response to venetoclax^[37]^. Additional research into cell metabolism may further elucidate details regarding these complex interactions and potential treatment targets.

### Richter transformation

Transformation of CLL contributing to venetoclax resistance is one of the less well-studied mechanisms of resistance. Recent analysis has shown that increased genetic instability during transformation can result in the development of mutations related to venetoclax resistance^[27]^. While the more common BCL2 mutation, Gly101Val, was not seen in the transformed population, a rarer mutation, Arg110dup, was seen in a low percentage (< 0.5%)^[18]^. Rates of Richter’s transformation vary by trial but were generally low [Table 1]. Currently, data on Richter transformation in CLL remains limited, but it is an area of ongoing investigation.

## METHODS TO OVERCOME RESISTANCE

While the first action after resistance to treatment is to alter treatment to another agent, researchers have identified several other avenues to combat resistance, including other formulations of bcl2 inhibitors, chimeric antigen receptor T (CART), BTK degraders, non-covalent BTK inhibitors, phosphoinositide 3-kinase inhibitors, and novel bispecific antibodies. Additionally, duration of treatment, fixed *vs.* continuous, may be instrumental in the development of resistance. An investigation into relapsed CLL patients treated with venetoclax and rituximab followed by venetoclax monotherapy found that among the durable responses (33 patients of which 14 remained on monotherapy and 19 stopped venetoclax), five-year estimates of ongoing response rate were similar, 71% (95%CI, 39-88) in continuous treatment *vs.* 79% (95%CI, 49-93) in the fixed duration group^[38]^. However, an analysis of single-agent venetoclax in CLL patients with prolonged follow-up found ongoing venetoclax treatment may be a driver of resistance, as activation of NF-kB with associated MCL1 expression was increased in all relapsed samples while on venetoclax therapy compared to off therapy^[39]^. This concept is further supported by a patient who achieved minimal residual disease on venetoclax with fixed treatment duration, and did not have increased NF-kB or other cell survival signaling^[39]^. Further, analysis of the MURANO trial with fixed duration combination treatment did not identify any mutations in *BCL2*, a known mechanism of resistance as discussed above^[40]^.

### BCL2 inhibitors/BH3 mimetic

Since the discovery of the BCL2 family of proteins involved in apoptosis, there has been an evaluation of BCL2-targeted agents. Obatoclax, a BH3 mimetic that antagonizes Mcl-1/Bcl-xL and Bcl-w but not BCL2, was evaluated in a phase I/II with bortezomib in relapsed refractory mantle cell lymphoma, but ORR was modest at 31% with myelosuppression and fatigue as the most common grade 3/4 adverse events^[41]^. Additionally, navitoclax, another BH3 mimetic, demonstrated 55% ORR when used with Rituximab for 12 weeks and 70% when used with Rituximab continuously until progression or intolerance compared to 35% ORR with rituximab alone in previously untreated CLL patients^[42]^. Unfortunately, significant thrombocytopenia limited widespread use as it was often dose-limiting^[43,44]^. Recently, Lisaftoclax, which selectively binds Bcl2 and prevents BCL2:BIM complexes allowing pore formation in mitochondria, demonstrated significant antitumor activity in preclinical trials^[45]^. This prompted progression to a phase I/II clinical trial in relapsed/refractory CLL patients with an ORR of 65% in the monotherapy group, 98% ORR in combination with acalabrutinib, and 87% in combination with Rituximab^[46]^. The average number of previous treatments was 2, with 12% of the patients progressing on BTK inhibitors and/or venetoclax^[46]^.

### Novel agents

In addition to BH3 mimetics, there has been an investigation into alternative targets with Bispecific antibodies/BiTE. As with venetoclax, the novel agents are explored across B-cell malignancies. Mosunetuzumab, a bispecific T cell engager targeting CD20/CD3, is under evaluation in Non-Hodgkin’s lymphoma (NHL) and CLL refractory to at least two lines of treatment in a phase I/II trial [NCT02500407] after promising results of a 60% CR in follicular lymphoma^[47]^. Other bispecific antibodies targeting CD20/CD3 are currently in various stages of clinical trials, namely odronextamab, glofitamab, epcoritamab, and plamotamab^[48-51]^. The most common serious adverse event across this drug class remains cytokine release syndrome^[48-51]^. At this time, these studies consist of mostly large B-cell lymphoma and follicular lymphoma patients, but in the future, the trials could be expanded to include refractory CLL patients.

Since the last decade, the implementation of CART has greatly impacted hematology and the treatment of hematologic malignancies. Initial evaluation of CART in CLL over a decade ago had a small sample size (2 patients), but on long-term follow-up, the patients continued to have a durable remission^[52,53]^. An analysis of several studies (15 studies, 160 patients) found decreased efficacy of CART in CLL patients with an average CR rate of 30% (0% to 67%) and suggested T-cell dysfunction as a potential rationale for the less robust response^[54]^. However, a large multicenter study of lisocabtagene maraleucel has recently shown rapid, deep, and durable responses in relapsed/refractory CLL patients after BTKi and venetoclax use^[55]^.

Additionally, there are ongoing clinical trials evaluating novel agents such as BTK degrader (NX-2127) and non-covalent BTK inhibitor (Pirtobrutinib) in relapsed/refractory patients^[56,57]^. Initial data on pirtobrutinib demonstrated promising results in BTK inhibitor refractory patients, with an ORR of 79% in patients (100 patients) who were refractory to both BTK inhibitors and *BCL2* inhibitors^[54]^. This success prompted a large phase III trial evaluating pirtobrutinib in the first line in CLL/SLL *vs.* ibrutinib and bendamustine + rituximab^[58,59]^. The combination of pirtobrutinib and venetoclax is currently in a phase II study in CLL patients in the first line with the primary endpoint of minimal residual disease after 15 cycles [NCT05677919]. Other non-covalent BTK inhibitors such as fenebrutinib and nemtabrutinib have also been investigated, but as both had limited success in phase I trials, their use in B cell malignancies was stopped^[60,61]^. Phosphoinositide 3-kinase inhibitors have some proven efficacy in CLL, namely idelalisib and duvelisib, with many others under various stages of investigation^[62-64]^. Combination treatments instead of single-agent therapy may be a method to overcome resistance, as previous CLL studies found that 50% of the patients became refractory to single-agent venetoclax after 2-3 years^[4,9]^.

## CONCLUSION

Since the widespread use of venetoclax, more and more has been discovered about intrinsic and extrinsic mechanisms of resistance. Genetic mutations in Bcl-2 are the most common form of venetoclax resistance but have only been reported in approximately 50% of resistant patients, although small sample size (15 patients)^[14]^. The less well-known forms of resistance, such as epigenetic modifications, alterations of oxidative phosphorylation, and Richter’s transformation, may play a larger role in resistance than we know and may become essential in future research of relapsed disease. Given this heterogeneity in resistance in both mechanisms and timeline of development, testing for resistance prior to venetoclax initiation is not warranted. However, there is some data supporting NF-kB expression as a potential biomarker for venetoclax resistance^[39]^, but more investigation is needed to determine its validity prior to widespread application.

Despite the extensive modes of venetoclax resistance, treatment is effective with both monotherapy and combination therapy^[4-10]^. Even refractory/relapsed CLL patients with poor prognostic factors, like 17p deletion, had durable responses to venetoclax in a phase 2 trial with 54% PFS at 24 months^[9]^. Interestingly, in a post-hoc analysis of the MURANO trial, dose reduction of venetoclax did not have a significant impact on PFS as long as treatment was not terminated^[65]^. The implication of dose reduction on resistance was not examined, but it would be beneficial to determine whether lower doses contribute to resistance as this would change management. Additionally, we need further analysis on fixed versus continuous treatment on the development of resistance as initial investigation supports fixed duration treatment in preventing at least certain types of resistance. Research into continuous *vs.* fixed treatment in combination treatments, particularly combination oral agents, could provide clarification.

Novel treatments like bispecific antibodies, BTK degraders, non-covalent binding BTK inhibitors, phosphoinositide 3-kinase inhibitors, and CART may provide the solution for relapsed/refractory patients, but their sequencing order in treatment remains to be determined, especially in CLL where the treated patient population is small. Given the relative novelty of bispecific antibodies, BTK degraders, non-covalent BTK inhibitors, and CART, we do not have long-term data on the impact on resistance. Retrospective analysis of the various clinical trials may provide some insights and should be an area for further research. Regardless of the potential resistance, we can conclude that venetoclax remains a cornerstone in the treatment of CLL.

## DECLARATIONS

### Copyright

© The Author(s) 2023.
